# The declining trend of HIV-Infection among pregnant women in Cameroon infers an epidemic decline in the general population

**DOI:** 10.1016/j.heliyon.2020.e04118

**Published:** 2020-06-11

**Authors:** Serge-Clotaire Billong, Joseph Fokam, Jean de Dieu Anoubissi, Cyprien Kengne Nde, Raoul Toukam Fodjo, Marinette Ngo Nemb, Yasmine Moussa, Arlette Lienou Messeh, Alexis Ndjolo, Jean-Bosco Nfetam Elat, the National HIV Drug Resistance Working Group

**Affiliations:** a Central Technical Group, National AIDS Control Committee, Yaoundé, Cameroon; b Faculty of Medicine and Biomedical Sciences, University of Yaoundé 1, Yaoundé, Cameroon; c National HIV Drug Resistance Working Group, Ministry of Public Health, Yaoundé, Cameroon; d Chantal BIYA International Reference Centre for Research on the Prevention and Management of HIV/AIDS, Yaoundé, Cameroon

**Keywords:** Cell biology, Virology, Evolutionary biology, Systems biology, Public health, Hematological system, Sentinel surveillance, Cameroon, Pregnant women, Population-based survey, HIV

## Abstract

**Background:**

HIV remains a generalised epidemic in Cameroon, with regular sentinel surveillance surveys (SSS) conducted among pregnant women to monitor the epidemiological dynamics, and for strategic policy making. Our main objective was to actualise data on HIV epidemiology, and compare the trends overtime among pregnant women versus data from the general population in Cameroon.

**Methods:**

Sentinel surveillance was conducted in 2016 among pregnant women in the 10 regions (60 sites) of Cameroon, targeting 7,000 first antenatal care (ANC-1) attendees (4,000 in urban; 3,000 in rural). HIV testing was done following the serial national algorithm at the National Public Health Laboratory. Results of 2016 were compared with 2009 and 2012 dataset, alongside reports from the general population; with p < 0.05 considered statistical significant.

**Findings:**

A total of 6,859 ANC-1 (97.99% sampling) were enrolled in 2016, with 99.19% (6,513/6,566) acceptability for HIV testing; similar to performances in 2009 and 2012 (>99%). National prevalence of HIV was 5.70% (389/6,819), similar between urban (5.58%) and rural (5.87%) settings. HIV prevalence among pregnant women declined significantly from 2009 (7.6%), 2012 (7.8%) to 2016 (5.7%), p < 0.0001; with a similar declining trend in the general population: from 2004 (5.5%), 2011 (4.3%) to 2017 (3.4%), p < 0.0001. Difference between SSS and the population-based survey was non-significant (r = 0.6; p = 0.285). Following geographical settings, HIV prevalence was higher in urban vs. rural settings from 2009-2012 (p < 0.0001), followed by similar rates in 2016. Early-age infection (15–24 years) decreased from 6.7% in 2009 to 3.4% in 2016, with remarkable declines in new infections within the age ranges 15–19 years (5.1%–1.57%) and 20–24 years (7.8%–4.39%).

**Interpretation:**

With high acceptability in HIV testing, the prevalence of HIV-infection through SSS indicates a declining but generalised epidemic among pregnant women in Cameroon. Of note, as the declining prevalence among pregnant women also reflects an epidemic reduction in the general population, SSS represents an efficient strategy to understand the dynamics of HIV epidemics in the general Cameroonian population, pending validation by periodic population surveys.

## 1 Introduction

The population of Cameroon was estimated to 22,709,892 inhabitants by end 2016 [1], and data on the burden of HIV epidemics revealed a reduction in the national prevalence from 2004 (5.5%), 2011 (4.3%) to 2017 (3.4%) following nationally-representative surveys including Health and Demographic Surveys (HDS) [2, 3, 4]. Similarly, HIV epidemiological updates, based on Estimations and Projections Package (EPP) and Spectrum that fit prevalence curves to surveillance and survey data [5], also revealed a decreasing epidemic in the general population (5.3% in 2009 to 3.8% in 2016) [5-7]. In spite of this progress, the country remains in a context of generalised HIV epidemic, which warrants regular surveillance for strategic information and impact at country-level [6, 7, 8, 9].

Epidemiological surveillance of HIV/AIDS provides verifiable information on the burden and distribution of infection, which serve as reference for effective planning, implementation, monitoring, and evaluation of the national AIDS prevention and treatment strategies [10]. The strengths of such surveillance systems rely on using the same design, populations and tools in assessing the dynamics of infection [9, 10]. Of note, assessment of HIV prevalence is generally based on HDS (conducted every half-decade and costly) [2, 3, 4], and on sentinel surveillance surveys [SSS] (conducted biennially and less costly) in sub-populations such as pregnant women attending antenatal care (ANC) [9, 10].

Though less representative of the general population, pregnant women represent a target that is easily accessible in the program, with 80–94% attending ANC in sub-Saharan Africa (SSA) [10]. In Cameroon, the overall rate of pregnant women attending ANC ranges from 75% (rural) to 94% (urban) [3], which underscores the significance of using this target population for purpose of HIV SSS [9, 10].

HIV SSS among Cameroonian pregnant women started in 1989 within the two major cities: Yaoundé and Douala [11]. Thereafter, the number of sentinel sites increased gradually: from six in 1993 (Yaoundé, Douala, Bamenda, Limbe, Bertoua and Garoua) to 27 from the 10 regions in 2000 [11]. These previous SSS showed an increasing burden of HIV infection among pregnant women between 1989 (0.5%) and 2000 (11%), followed by a decline in 2002 (7.3%) [11]. To ease the implementation of SSS in resource-limited settings (RLS), the World Health Organisation (WHO) recommended as of 2006 the use of PMTCT data for purpose of SSS in high-priority (i.e. high HIV burden) countries like Cameroon [9, 10]. In this new era, Cameroon reported a prevalence of 7.6% (2009) and 7.8% (2012) [10,11]. This new approach presents several advantages: (a) respect of participant autonomy, (b) HIV result delivery and linkage to care if seropositive, (c) scale-up of coverage in SSS/PMTCT, and (d) easy monitoring-evaluation system [12].

However, placing the current SSS strategy in the context of other epidemiological surveillance approaches, such DHS or EPP Spectrum reporting systems, would provide greater interpretation of the national AIDS epidemics, dynamics of infection, and guide on recommendations for priority interventions based on high quality evidence in a context of limited resources [5, 9, 10]. In this prospect, we therefore hypothesized that, using the current HIV SSS approach will not only inform on the trends in HIV infection among pregnant women, but will also depict the dynamics of infection in the general population, for setting-up national programmatic interventions.

Our objectives were to: (a) update the epidemiological burden of HIV among pregnant women in Cameroon during the year 2016, (b) evaluate the trends of HIV infection among pregnant women in the SSS, and (c) compare the dynamics of HIV between the SSS and population-based surveys.

## 2 Methods

### 2.1 Study design

A cross-sectional study was conducted among pregnant women attending their first ANC visit (ANC-1) in 60 sentinel clinical sites of the 10 regions of Cameroon in 2016, with comparison to 2012 and 2009 SSS and population-based dataset. Of note, 2016, 2012 and 2009 represent the period whereby sentinel surveillance sruveys were conducted using PMTCT data in the country.

Based on WHO strategy for HIV SSS among pregnant women [9], a systematic sampling method was done in two stages: (1) selection of sentinel sites, and (2) selection of pregnant women in each study sentinel site. Briefly, study sites were selected by based on: (a) representativeness at regional level, (b) geographical location in each region (urban or rural), (c) availability of PMTCT services and data management system, (d) functionality of the site (staff and materials for ANC/PMTCT, laboratory services and a cold chain), and (e) site willingness for participation into the study. Selected sites were from health facilities of the primary, secondary and tertiary healthcare levels. At study site, pregnant women were enrolled based on the following criteria: (a) aged from 15 to 49 years, (b) attending ANC-1, (c) registered in the study facility, and (d) consent for participation.

The minimum sample size required was calculated as per the WHO sampling strategy [9, 10], using the prevalence obtained (P) for urban and rural settings of each region during the previous survey conducted in 2012 [12], with 95% confidence interval α = 0.05 (Z_α/2_ = 1.96), precision with an error rate i = 0.03 (the choice of 0.03 was used for greater estimates to the target population).

$$n=\frac{z_{\alpha /2}^{2}}{i^{2}}P\left(1-P\right)$$

The 10% increment on sample size was intended to overcome cases of rejection due to refusal to consent, possible errors in sampling (haemolysis, labelling, processing, poor storage conditions). Thus, a total of 300 and 400 participants were required respectively for rural and urban settings per region, giving an overall target of 7,000 participants (3,000 rural and 4,000 urban) from the 10 regions of Cameroon (HIV proportion used in each region is provided in Table 1).

**Table 1:** Minimum sample size required for urban and rural settings of each region [12, 13, 14].

No	Regions	Prevalence observed in the 2012 HIV-SSS		Minimum number of pregnant women to be enrolled		Number of pregnant women to be enrolled (i.e. 10% round up)	
		Rural	Urban	Rural	Urban	Rural	Urban
1	Adamawa	6.98	4.85	277	197	305	217
2	Centre	9.43	13.88	365	510	401	561
3	Far-North	0.68	7.05	29	280	32	308
4	East	10.7	7.26	408	287	449	316
5	Littoral	9.59	10.67	370	407	407	448
6	North	3.01	5.61	125	226	137	249
7	North-West	7.95	9.44	312	365	344	401
8	West	6.12	4.02	245	165	270	181
9	South-West	10.03	11.05	385	420	424	461
10	South	11.84	8.41	446	329	490	362

SSS: Sentinel Surveillance Survey.

### 2.2 Procedures

**Selection of the reference laboratory**: The National Public Health Laboratory (NPHL) was selected as the national reference laboratory (NRL) for the survey, based on its participation in the Centers for Disease Control and Prevention (CDC)/the United States President's Emergency Plan for AIDS Relief (PEPFAR) HIV proficiency testing as well as satisfactory results of External Quality Control in HIV testing (100% positive concordance and 100% negative concordance) conducted on a panel of clinical samples from the Chantal BIYA International Centre for Research on HIV/AIDS prevention and management (CIRCB), serving as the quality assessment laboratory for the NPHL.

**Staff training and field preparedness**: For purpose of quality assurance, standard sheets were designed and validated for the clinical site assessment, laboratory assessment, SSS data collection tools, and sample shipment. To ensure reliability in data collection and mastering of the standard operational procedures (SOP) for HIV testing in all sites, staffs (public health experts, nurses or mid-wives and laboratory technicians) were trained on the study goals and SOPs, with a technical assistance from the WHO, CDC/PEPFAR, the Joint United Nations Programme on HIV/AIDS (UNAIDS), NRL, CIRCB, and other partners. For a total of 120 trainees, three training pools were organised nationwide as follows: Pool A (Adamawa region, Far-North region and North region); Pool B (Centre region, East region and South region); and Pool C (Littoral region, North-West region, West region and Southwest region).

**Collection of sociodemographic data**: At each sentinel site, a standard questionnaire was used by the nurse/mid-wife for each eligible pregnant woman to collect specific information: region, geographical location (urban/rural), knowledge of HIV status and history of HIV screening, (age, level of education, parity, marital status, gestational age, profession, etc). A code, linking the questionnaire to the HIV laboratory request form, was used for purpose of confidentiality and for retracing cases eligible for treatment. Completeness of questionnaire was verified before submission at the central-level (NRL).

**Collection of blood samples**: From each consenting pregnant women, a total of 5ml of whole blood was collected by venepuncture on EDTA tubes by trained phlebotomists. Plasma was separated from whole blood, stored at 0–8 °C, and then shipped at the NRL for HIV testing. Once at the NRL, plasma samples were stored at -80 °C until processing for HIV testing.

**Figure 1:**
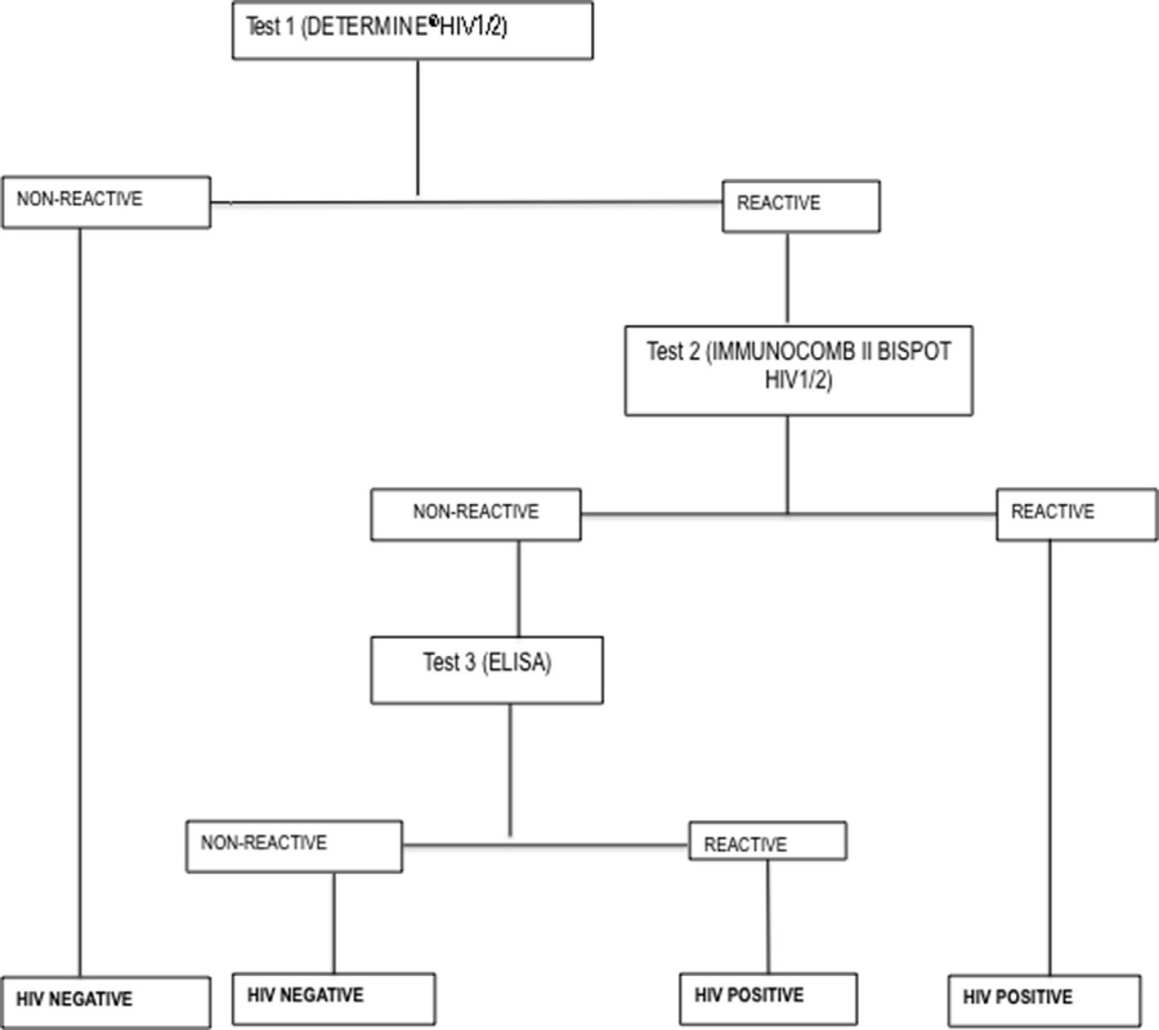
Algorithm for HIV testing during the sentinel surveillance survey. Legend. ELISA: Enzyme linked immunosorbent assay.

**HIV testing algorithm**: A serial algorithm for HIV testing was used at the NRL, in conformity with the national algorithm for voluntary HIV testing (Figure 1). Briefly, all samples were processed with the first test, Determine HIV1/2 (*Abbott, Minato-ku-Tokyo, Japan*). Samples reporting a non-reactive result with Determine HIV1/2 were considered HIV seronegative; for samples reactive to Determine HIV1/2, IMMUNOCOMB II BISPOT HIV1/2 *(OraSure Technologies, Inc, Bethlehem, Pennsylvania)* was performed; samples that were confirmed reactive were considered HIV seropositive, while in case of a discordant result between test 1 (reactive to Determine HIV1/2) and test 2 (non-reactive to IMMUNOCOMB II BISPOT HIV1/2), an enzyme-linked immunosorbent assay (ELISA) was used as tie-breaker. Conclusive results (HIV seropositive or seronegative) were then reported on the surveillance form through the unique identifier of each participant at the NRL [15].

### 2.3 Data management and statistical analysis

Validated datasheets were entered in the Census and Survey Processing System, version 6.0 (CSPro.v6) to minimise errors in data entry. Data cleaning and analysis were performed using STATA®/IC12.0 (College Station, Tx: stataCorp LP).

HIV seropositivity was calculated and expressed in percentages; comparaison of HIV seropositivity rate between region, geographical location and age, was done using chi square test.

As the study methodology was the same in all the study periods, the three datasets were appended and analysed as one. Trends of HIV infection in 2009, 2012 and 2016 were compared between the SSS and the respective population-based surveys; with p-value <5% considered statistically significant. Spearman was used to evaluate the strength of correlation; with X referring to SSS-data and Y to the respective population-based surveys (http://www.socscistatistics.com/tests/spearman/Default2.aspx).

### 2.4 Ethical considerations

Ethical clearance for the study was obtained from the Cameroon National Ethics Committee for Research on Human Health (Ref N^o^ 879/CE/CNERSH/SP). Each pregnant woman provided a verbal consent prior to enrolment; confidentiality was ensured through the use of specific identifiers; HIV test results were free of charge for all pregnant women; HIV-positive cases were treated free of charge using the current PMTCT option B+ national guidelines, with linkage to care using psycho-social agents wherever necessary; data integrity was ensured by double check (on site and at central-level) and access to data was restricted using an encrypted password.

### 2.5 Role of the funding sources

The sponsors, namely the World Health Organization, CDC PEPFAR and Global Fund provided funds and technical assistance for the 2009, 2012 and 2016 surveys respectively. The sponsors had no direct role in study design, data collection, analysis, interpretation, technical reporting, scientific writing of the manuscript, or decision to submit the manuscript for publication. The corresponding authors had full access to all data, and responsibility for submission for publication.

## 3 Results

### 3.1 Sociodemographic profile of pregnant women enrolled in 2016

Out of the 7,000 expected targets, 6,859 pregnant women were successfully enrolled, giving an overall sampling of 97.99%. Twelve were excluded due to age following data verification at central-level (i.e. 11 aged <15 years and one older than 49 years), giving a total of 6,847 participants in the final dataset. According to geographical locations, sampling coverage was 98.15% (3,926/4,000) and 97.36% (2,921/3,000) in urban and rural settings respectively (Table 2).

**Table 2:** Distribution of pregnant women enrolled by region and by geographical locations.

Region	Urban		Rural		Overall	
	Number of pregnant women	Sampling coverage (%)	Number of pregnant women	Sampling coverage (%)	Number of pregnant women	Sampling coverage (%)
Adamawa	475	118.75	202	67.33	677	96.71
Centre	541	135.25	137	45.66	678	96.86
East	317	79.25	380	126.66	697	99.57
Far-North	327	81.75	355	118.33	682	97.43
Littoral	448	112.00	169	56.33	617	**88.14∗**
North	406	101.5	312	104.00	718	102.57
North-West	353	88.25	323	107.67	676	96.57
South	402	100.5	271	90.33	673	96.14
South-west	246	61.5	472	157.33	718	102.57
West	411	102.75	300	100.00	711	101.57
Total	3926	98.15	2921	97.36	6847	97.81

In bold∗ is region with lowest enrolment performance.

Median age was 26 [IQR: 21–30] years; more than half (57.06%) were aged ≥25 years, with a similar trend between urban (59.36%) and rural (53.96%) settings; and 15.04% were adolescents aged 15–19 years (Table 3).

**Table 3:** Distribution of pregnant women by age.

Age range (years)	Geographical location					
	Rural		Urban		Overall	
	Number	%	Number	%	Number	%
<25	**1332**	**46.04**	**1586**	**40.64**	**2918**	**42,94**
15–19	519	17.94	503	12.89	1022	15.04
20–24	813	28,1	1083	27,75	1896	27,9
≥25	**1561**	**53.96**	**2317**	**59.36**	**3878**	**57.06**
25–29	732	25.3	1103	28.26	1835	27
30–34	493	17.04	789	20.22	1282	18.86
35–39	261	9.02	355	9.1	616	9.06
40–44	66	2.28	65	1.67	131	1.93
45–49	9	0.31	5	0.13	14	0.21
Total	**2893**	**100.00**	**3903**	**100.00**	**6796**	**100.00**

In bold are high proportions (≥40%).

According to educational level, 47.40% had attended secondary education (51.35% in urban and 42.08% rural settings), 13.59% had attended higher education, while 13.97% have never been provided with schooling. According to professional occupation, the majority (50.50%) were housewives, followed by students (17.32%), traders (6.13%), tailors (5.93%), farmers (5.13%), teachers (4.92%), hairdressers (3.77%), and others. According to marital status, the majority (78.03%) were married or living with a partner, 21.69% were single, and very few were widows or had divorced.

### 3.2 Acceptability for HIV testing

Of the 6,847 pregnant women enrolled, the acceptability rate for HIV testing was 99.59% (6819) at national-level. According to geographical locations, the acceptaibility rate varied from 99.46% (3,905/3,926) in urban to 99.76% (2,914/2,921) in rural settings.

### 3.3 HIV prevalence

Among pregnant women tested for HIV, the overall prevalence of HIV was 5.7% (389). Of note, 4 HIV-1 positive cases were also reactive to HIV-2 (detected by Immunocomb II Bispot HIV1/2), suggesting a coïnfection rate of HIV-1 and HIV-2 of 0.06% in the study population.

According to geographical locations, HIV prevalence ranged from 5.58% (218/3,905) to 5.87% (171/2,914) in urban and rural settings respectively, without any significant difference (p = 0.6186). However, according to regions, HIV prevalence varied significantly from 2.65% (North region) to 9.74% (East region); with 3.41% (115/3367) in the five low-prevalence regions versus 7.90% in the five high-prevalence regions, p < 0.0001. Of note, the lowest HIV prevalences were found in the Northern part of Cameroon (North: 2.65%; Adamawa: 3.25% and Far-North: 3.68%), while the highest HIV prevalences were reported in the East (9.74%), Centre (9.51%), and Southwest (9.07%) regions (Table 4).

**Table 4:** HIV prevalence by region and by geographical locations.

Region	Urban		Rural		Overall	
	Number of pregnant women	HIV prevalence	Number of pregnant women	HIV prevalence	Number of pregnant women	HIV prevalence
Adamawa	475	3.16	202	3.47	677	3.25
Centre	541	**9.70**	137	**8.76**	678	**9.51**
East	317	**9.68**	380	**9.79**	697	**9.74**
Far-North	327	4.91	355	2.55	682	3.68
Littoral	448	3.14	169	4.73	617	3.58
North	406	3.95	312	0.96	718	2.65
Northwest	353	**5.95**	323	**6.81**	676	**6.36**
West	402	2.44	271	**6.02**	673	3.95
South	246	**5.01**	472	**5.19**	718	**5.08**
Southwest	411	**9.76**	300	**8.70**	711	**9.07**
Total	**3905**	**5.58**	**2914**	**5.87**	**6819**	**5.70**

In bold are high HIV prevalences (≥5%).

According to geographical locations within each region, only the West region showed a significant disparity in HIV prevalence between the urban (2.44%) and the rural setting (6.02%), p < 0.0001. The other 9 regions had similar distributions of HIV prevalence between the urban and rural settings (Table 4).

### 3.4 National trends of HIV infection between the years 2009 and 2016

⁃ Trends of HIV prevalence among pregnant women 2009-2016

**Figure 2:**
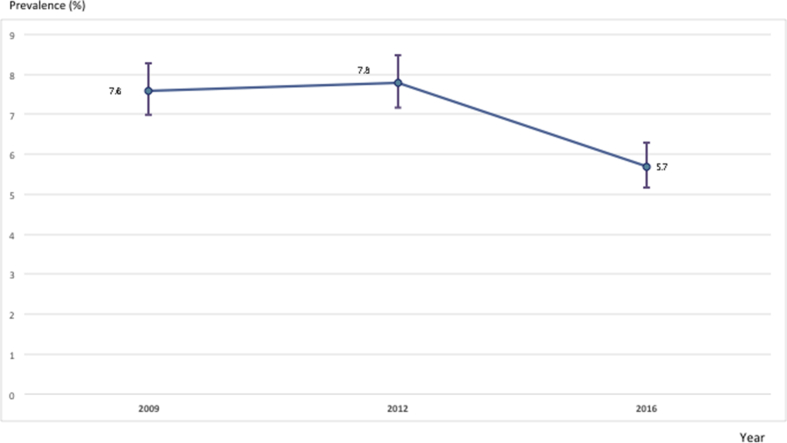
Evolution of the national HIV prevalence among pregnant women from 2009 to 2016. Legend. Numbers on the graph represent percentage per year.

From 2009 to 2016, evolution of the national HIV prevalence among pregnant women in the SSS showed a significant decline from 7.6% to 5.7%, p < 0,0001 (Figure 2).

**Figure 3:**
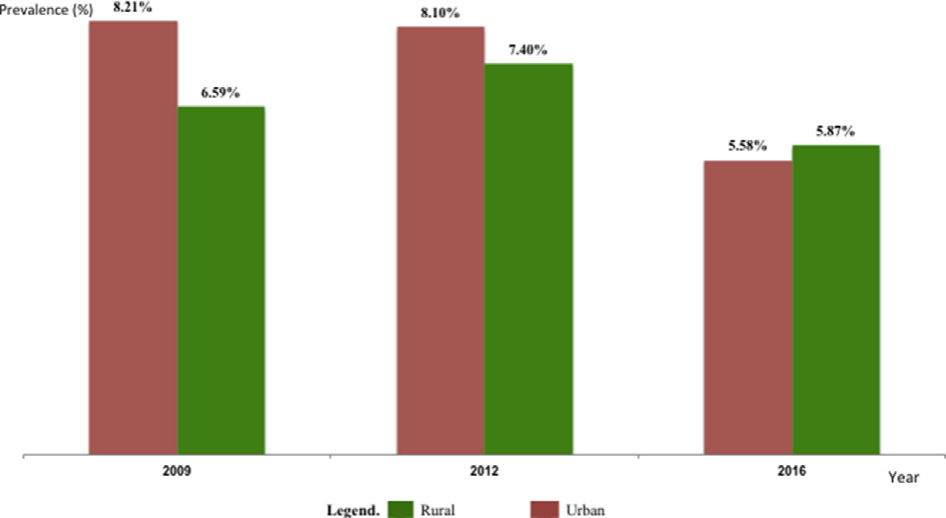
Evolution of HIV prevalence among pregnant women between urban and rural settings from 2009-2016.

According to geographical locations overtime, HIV prevalence was significantly higher in urban (8.21–8.1% in urban versus 6.59–7.4% in rural) settings between 2009-2012 respectively (p < 0.0001), followed by a similar prevalence between the two locations in 2016 (5.58% in urban versus 5.87% in rural, p = 0.615), indicating higher incidence of HIV among pregnant women in rural settings (Figure 3).

**Figure 4:**
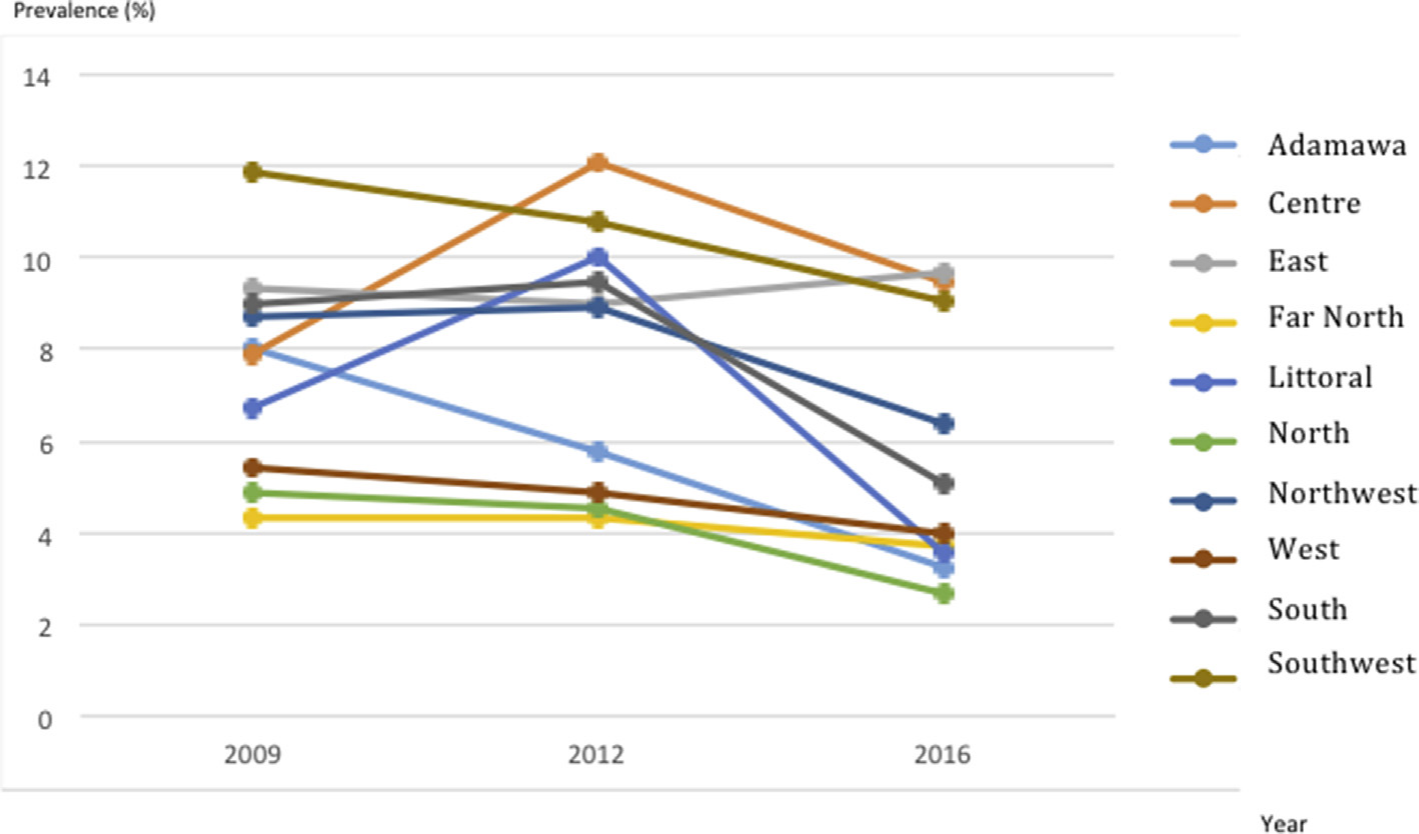
Evolution of regional HIV prevalence among pregnant women from 2009 and 2016.

According to regions, HIV prevalence varied significantly (p < 0.0001). Specifically, from 2009 to 2016 showed a decreasing trend in the West, Southwest, North, Adamawa and Far-North regions; an increasing trend (i.e. high incidence among pregnant women in this region) in the East region; while a fluctuating prevalence was observed in the Centre, Littoral, South, and Northwest regions (Figure 4).

**Figure 5:**
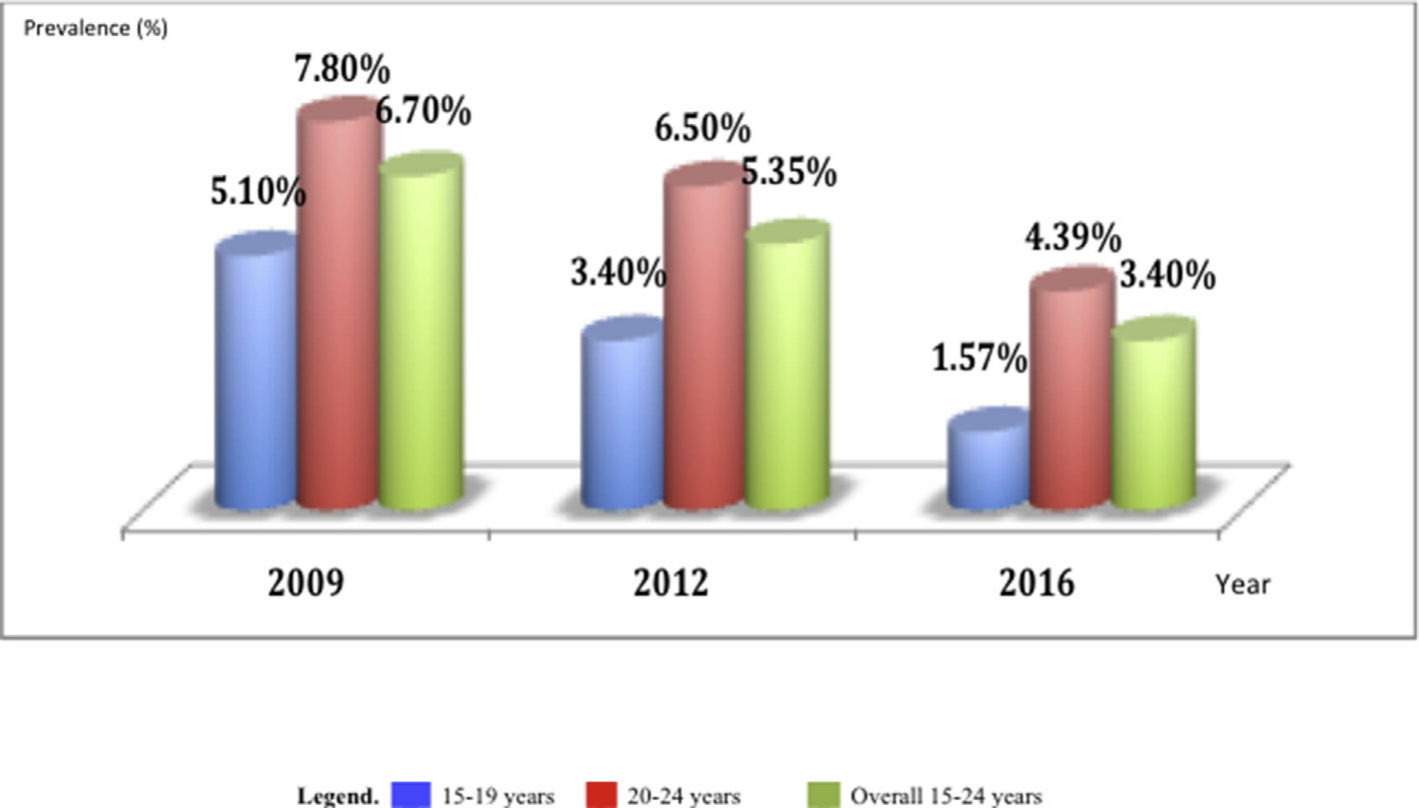
Evolution of early HIV infection among pregnant women between 2009 and 2016.

According to rates of new HIV infection, assessed at early sexual age (15–24 years), a declining prevalence was observed overtime, ranging from 6.7% (2009) to 3.4% (2016). Furthermore, similar declining trends were also observed between 2009 and 2016 in the subset of participants aged 15–19 years (from 5.1% to 1.57%) and 20–24 years (from 7.8% to 4.39%), thus indicating a decreasing rate of early infections among pregnant women (Figure 5).

⁃ Comparative trends of HIV infection from SSS versus population data 2009-2016

From 2009 to 2016, the prevalence of HIV in the general population using population-based survey showed a dropdown, ranging from 5.5% in 2009, 4.3% in 2012, to 3.4% in 2016; p < 0.001.

**Figure 6:**
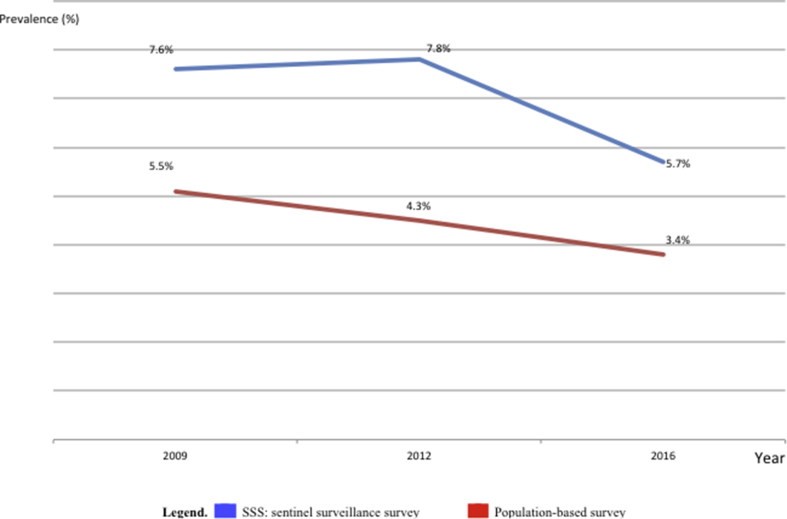
Comparison of HIV trends between SSS and population-based survey. Legend. SSS: Sentinel Surveillance Survey.

A comparison of trend between HIV-SSS and HIV-population-based data in the same years revealed similar declining trends in HIV prevalence (Figure 6), with a moderate correlation (r = 0.6). These revealed an overall epidemiological decline of -1.9% (using SSS) versus -2.1% (using population-based data), with a statistically non-significant difference between both approaches (p = 0.285).

## 4 Discussion

In a context of HIV epidemic, pregnant women constitute a key target in depicting evolutionary trend of infection in the general population, and serving for timely actions in prevention and treatment programs in RLS [9, 10]. The current study, focused on updating HIV surveillance data and comparing epidemiological trends among pregnant women to those obtained from the general population, would therefore contribute in defining the relevance of SSS in monitoring HIV infection towards modelling of interventions in RLS. In implementing this study, HIV tests used were selected based on excellent diagnostic performance (sensitivity, specificity) reported locally on HIV types 1 and 2 [16], thus ensuring reliability in our serological results.

Our findings revealed 97.99% (6,859) sampling for HIV testing, SSS generates data that are representative of the target population of pregnant women [10, 11]. With 99.19% acceptance for HIV testing, pregnant women strongly adhere to routine screening. This high maternal acceptability to HIV testing was largely due to adequate counselling on PMTCT option B+. Thus, settings with poor acceptability to HIV testing (97.78% in the southwest region) require investigations on determinants (local culture, perception of HIV/AIDS, etc) to further reduce risks of HIV transmission nationwide [6, 7, 11, 12]. Moreover, adolescent pregnancy (15%), similar to previous reports (18% in 2012 nationwide; 26% in Brazil), reveals persistent unsafe sexual practices that require preventive measures against HIV acquisition [11, 13, 17]. Henceforth, integrating local culture and possible age-dependent factors into the current counselling strategy might improve coverage, and adherence throughout the PMTCT cascade [17, 18].

With 5.7% national prevalence of HIV, similar between urban and rural settings (p = 0.6186), pregnant women share similar risks of infection regardless of residential area. However, regions with the highest rate (9.7% East; 9.5% Centre; 9.05% Southwest) also reflected the HIV prevalence in the general population of that region (6.3% East; 6.3% Centre; 5.7% Southwest) [8]. These findings underline these regions for priority interventions [13, 18]. Discrepant prevalence of HIV was observed only in the South region (5.07% in SSS and 7.2% in the general population), probably due to population migration across the border with the respective neighouring countries [7, 8]. As previously reported, interventions in these regions with high HIV burden should focus on women with history of multiple pregnancies, advanced age, with a primary or secondary level of education, divorced and/or widows [13, 14].

Interestingly, the very low rate of reactivity to HIV-2 (0.06%) among pregnant women (4 cases) underscores the low risk of HIV-2 vertical transmission, and the scarcity HIV-2 in the general population, as reported locally by Njouom *et al.* at 0.83% (4) among 480 selected samples in Cameroon [16]. Rate of HIV-2 was higher among pregnant women in Mauritanie (6.5%), in accordance with the epidemiology of HIV-2 in West Africa [17].

Regarding HIV infection from 2009 to 2016, there is a significant declining trend in the population of pregnant women in Cameroon (from 7.60% to 5.7%, p < 0.0001), which is in line with the dropdown of epidemic reported in the national HDS (from 5.5% in 2004 to 4.3% in 2011) [2,3], followed by 3.4% in 2017 [4]. Thus, pending report of the national population-based HIV assessment, our analysis reveals that the declining trend in SSS (-1.9% reduction) is closely similar to the estimated dropdown in the general population (-2.1% reduction). This therefore reassures our surveillance model as an efficient strategy to monitor the programme performance in HIV prevention and treatment, which in turn would be cost-effective for implementation in situation of limited resources [9, 19]. Of note, this declining rate of HIV infection could be attributed to efforts linked to the increasing treatment coverage and prevention interventions. These indications are supported by the very low rate of HIV infection among adolescents (1.57%), which in turn represent case of new infection, translated as a low rate of HIV incidence in the general population [17, 18].

Following multivariate analyses, efforts in reducing significantly cases of new infection should target mainly women living in specific regions (Centre, South, Southwest, East, Northwest and Adamawa), and specifically those with advanced age (>24 years old), likely due to multiparity (translating multiple risky behaviours/exposure to HIV) in this age range [6, 7, 17, 18, 19].

For translational application at global-level, analysis of such epidemiological trends should be encouraged in other African countriers where HIV prevalence can be obtained from ANC and HDS [20, 21].

Major strengths of our study are: (a) the representativeness of sample with respect to the target population of pregnant women, (b) the high acceptability in HIV testing, (c) the use of a NRL for testing, and (d) the reliability of tests used for HIV screening [11, 12, 13, 14]; thus underlying the significance of our findings for evidence-based interventions as part of the national AIDS prevention and treatment strategies. A systematic review and/or meta-analysis, on the impact of PMTCT surveillance in identifying priority interventions for HIV prevention, would be of great importance.

Study limitations may entail the inability: (a) to further characterise the potential cases of HIV-2, and (b) to ascertain non-adherence to PMTCT and associated factors. Furture studies addressing these issues will generate evidence for a greater surveillance.

## 5 Conclusion

With ~98% sampling and 99% acceptance to ANC HIV testing, SSS is a reliable approach in appraising the prevalence of HIV-infection among pregnant women. More importantly, the concordance in the trends of HIV-prevalence between SSS and HDS underscores the utility of SSS as a timely and cost-effective strategy in understanding the dynamics of infection in the general population and in defining priority areas for programmatic interventions, pending validation by periodic population surveys. In countries with similar features like Cameroon, interventions toward a continuous epidemic decline may consider the geographical locations and advanced age.

## Declarations

### Author contribution statement

Serge-Clotaire Billong, Joseph Fokam: Conceived and designed the experiments; Performed the experiments; Analyzed and interpreted the data; Wrote the paper.

Jean de Dieu Anoubissi, Cyprien Kengne Nde, Raoul Toukam Fodjo, Alexis Ndjolo, Jean-Bosco Nfetam Elat: Conceived and designed the experiments; Performed the experiments; Analyzed and interpreted the data.

Marinette Ngo Nemb, Yasmine Moussa, Arlette Lienou Messeh: Performed the experiments; Analyzed and interpreted the data.

### Funding statement

This work was supported by the Global Fund; 10.13039/100009054CDC PEPFAR; 10.13039/100004423World Health Organization.

### Competing interest statement

The authors declare no conflict of interest.

### Additional information

No additional information is available for this paper.
